# Synergistic Effects of Concurrent Blockade of PI3K and MEK Pathways in Pancreatic Cancer Preclinical Models

**DOI:** 10.1371/journal.pone.0077243

**Published:** 2013-10-09

**Authors:** Hua Zhong, Cesar Sanchez, Dirk Spitrzer, Stacy Plambeck-Suess, Jesse Gibbs, Williams G. Hawkins, David Denardo, Feng Gao, Robert A. Pufahl, Albert C. Lockhart, Mai Xu, David Linehan, Jason Weber, Andrea Wang-Gillam

**Affiliations:** 1 Division of Oncology, Department of Medicine, Washington University in St. Louis, St. Louis, Missouri, United States of America; 2 Department of Hematology-Oncology, School of Medicine, Pontificia Universidad Catolica de Chile, Santiago, Chile; 3 Department of Surgery, Washington University in St. Louis, St. Louis, Missouri, United States of America; 4 Siteman Cancer Center, Washington University in St. Louis, St. Louis, Missouri, United States of America; University of L'Aquila, Italy

## Abstract

Patients with pancreatic cancer have dismal prognoses, and novel therapies are urgently needed. Mutations of the KRAS oncogene occur frequently in pancreatic cancer and represent an attractive target. Direct targeting of the predominant KRAS pathways have been challenging and research into therapeutic strategies have been now refocused on pathways downstream of KRAS, phosphoinositide 3-kinase (PI3K) and mitogen-activated protein kinase (MAPK [MEK]). We hypothesized that concurrent inhibition of the PI3K and MEK pathways would result in synergistic antitumor activity, as it would circumvent the compensatory feedback loop between the two pathways. We investigated the combined effect of the PI3K inhibitor, GDC0941, and the MEK inhibitor, AZD6244, on cell viability, apoptosis and cell signaling in a panel of pancreatic cancer cell lines. An *in vivo* analysis was conducted on pancreatic cancer xenografts. While BxPC-3 (KRAS wild type) and MIA PaCa-2 (KRAS mutated) cell lines were sensitive to GDC0941 and AZD6244 as single agents, synergistic inhibition of tumor cell growth and induction of apoptosis were observed in both cell lines when the two drugs were combined. Interestingly, phosphorylation of the cap-dependent translational components, 4E-binding protein (p-4E-BP1) and S6 was found to be closely associated with sensitivity to GDC0941 and AZD6244. In BxPC-3 cell xenografts, survival differences were observed between the control and the AZD6244, GDC0941, and combination groups. Our study provides the rationale for concurrent targeting of the PI3K and MEK pathways, regardless of KRAS status, and suggests that phosphorylation of 4E-BP1and S6 can serve as a predictive biomarker for response to treatment.

## Introduction

Pancreatic cancer is the fourth leading cause of cancer-related deaths in men and women in the United States. An estimated 43,140 people were diagnosed with and 36,800 died of pancreatic cancer in 2013 [1]. The lack of screening methods and effective therapeutic agents make detecting and treating pancreatic cancer a difficult problem. While targeted agents have become the mainstream for other types of cancer, at present, only the epidermal growth factor receptor inhibitor erlotinib has gained approval from the Food and Drug Administration for the treatment of pancreatic cancer [2]. Unfortunately, the clinical utility of erlotinib is largely limited due to its rather modest clinical benefit, reflecting a continued urgency to develop targeted agents in pancreatic cancer.

The presence of a KRAS mutation is seen in 30% of premalignant lesions [3] and in up to 90% of pancreatic cancer tumor specimens [4], suggesting that the KRAS mutation is the predominant known feature of pancreatic cancer molecular pathogenesis. KRAS is a GTPase, and it converts extracellular signals into intracellular signals by cycling between the active (RAS-GTP) and inactive (RAS-GDP) states. Mutated KRAS results in constant activation of the RAS pathway by locking RAS into the active GTP-binding state and further triggering multiple downstream signaling pathways including cell proliferation, apoptosis, differentiation, and survival [5]. Direct targeting of KRAS has not been successful in patients with pancreatic cancer [6], so current research efforts have refocused on two downstream pathways, the phosphatidylinositol 3-kinase (PI3K)/AKT pathway [7] and the RAF/MEK pathway [8,9].

Because cell signaling networks are complex, simply blocking one mediator is unlikely to result in a significant clinical response, unless the genetic alternation renders the targeted “effector” to be an oncologically driven event. This is hardly the case in KRAS downstream pathways, illustrated by the exceedingly low incidence of PIK3CA or BRAF mutations in pancreatic tumors [10]. Therefore, it has been hypothesized that concurrent blockade in two parallel pathways such as PI3K and MEK will significantly increase the chance for success in achieving a clinically relevant response. Indeed, synergistic anti-tumor effects have been observed when PI3K/AKT and MEK pathways are both inhibited in preclinical tumor models [11], including a KRAS mutated lung cancer model [12].

GDC0941 is an oral agent developed to inhibit all four class І PI3K isoforms [13]. It has dose-dependent anti-tumor activity against glioblastoma and human ovarian cancer xenografts [14]. GDC0941 has shown promising anti-tumor activity in the preclinical setting, and it is currently being tested in early phase clinical trials [14]. AZD6244 is a potent, selective secondary generation MEK1/2 inhibitor, which inhibits MAPK/ERK in an ATP-uncompetitive fashion [15]. Along with other MEK inhibitors, AZD6422 is currently in early phase clinical trials [16-18]. Preclinical evaluations of combining a PI3K/AKT inhibitor and a MEK inhibitor in pancreatic cancer are emerging [19], and our study confirms that a synergistic effect occurs when blocking these two pathways. Moreover, we have further illustrated that the benefit of concurrent blockade is not KRAS genotype limited. Additionally, our study shows that the translation process, in particular, activation of 4E-binding protein 1 (4E-BP1) and S6 seems to be associated with the pancreatic cancer cells’ phenotypic response toward the inhibitors.

## Materials and Methods

### Cell Culture and Inhibitors

Pancreatic cancer cell lines, BxPC-3 (KRAS wild type), MIA PaCa-2 (KRAS mutant), PANC-1 (KRAS mutant) and Capan-2 (KRAS mutant) were obtained from American Type Culture Collection (Manassas, VA, USA) and cultured in a growth medium of either DMEM (PANC-1, MIA PaCa-2), RPMI-1640 (BxPC-3) or McCoy’s 5A medium (Capan-2) supplemented with 10% fetal bovine serum, 100 units/ml penicillin, 100 µg/ml streptomycin and 1mM sodium pyruvate at 37°C in a humidified atmosphere containing 5% CO_2_. The PI3K inhibitor GDC0941 and MEK inhibitor AZD6244 were purchased from Selleck Chemicals LLC (Houston, TX, USA) and dissolved in dimethylsulfoxide. Both inhibitors were stored at -20°C.

### Cell Viability Assay

Pancreatic cancer cells lines were seeded at a density of 3,000 cells per well in a 96-well microtiter plate in growth medium and allowed to adhere overnight. GDC0941 or AZD6244 dose-response was determined by treating the pancreatic cancer cells lines with 5 concentrations of the drugs based on a 10-fold dilution series. Cell viability was assessed 72 hours later by Alamar Blue (Invitrogen, NY, USA) (570λ _Ex_/580λ_Em_) with a fluorescent microplate reader, and expressed as a percentage of drug-treated cells relative to control (no drug) cells. Data were analyzed using GraphPad Prism 5 software (GraphPad Software, Inc. San Diego, CA, USA), and the dose response curve was used to calculate the concentration of drug resulting in 50% inhibition of cell viability (IC_50_) using a four parametric logistical model. All assays were repeated five times.

For drug combination studies, the synergistic effect was assessed by the combination index (CI), according to the method of Chou & Talalay wherein synergism is defined as CI < 1, while antagonism is CI > 1, and an additive effect is considered as CI = 1 [20]. Cell lines were treated with GDC0941, AZD6244 or a combination of GDC0941 and AZD6244, and the number of viable cells was used to calculate the CI values using CalcuSyn software (Biosoft, Cambridge, United Kingdom).

### Apoptosis Assay

Approximately 2×10^5^ cells were seeded in 6-well plates for 24 hours. Cells were then treated with various concentrations of GDC0941, AZD6244 or a combination of GDC0941 and AZD6244 for 72 hours. Cells were harvested with 0.25% trypsin, washed with phosphate buffered saline (PBS), and collected together by centrifugation. The cells were then stained with the Annexin V-FITC and PI solution (BD Biosciences, San Diego, CA, USA) according to the manufacturer’s instructions and were analyzed with a FAC scan flow cytometer (Becton Dickinson, San Diego, CA, USA). All experiments were performed in triplicate.

### Western Blot Assay

Cells were seeded in 6-well plates for *in vitro* analyses. When cells became 70-80% confluent, they were incubated with GDC0941, AZD6244 or both GDC0941 and AZD6244 for 24 hours. Then the cells were washed with cold PBS, and lysed in RIPA buffer (50 mM Tris-HCl pH 7.4, 150 mM NaCl, 1% Triton x-100, 1% sodium deoxycholate, 0.1%, SDS, 1 mM EDTA, protease inhibitors). The supernatants were collected after sonification and centrifugation, and equal amounts of proteins were electrophoresed through 4-12% Bis-Tris Gels and transferred to nitrocellulose membranes (Invitrogen, Carlsbad, CA, USA). Membranes were blocked with 5% non-fat milk following overnight incubation with the appropriate primary antibodies at 4°C. All primary antibodies were incubated in 5% bovine serum albumin (Sigma-Aldrich, St. Louis, MO, USA). The membranes were washed 3 times to remove unbound antibody and then incubated with a horseradish peroxidase-conjugated secondary antibody for 1 hour at room temperature. Membranes were treated with enhanced chemiluminescence reagents according to the manufacturer’s protocol (GE Healthcare Life Science, PA, USA). Primary antibodies included anti-phospho-AKT (Ser473), anti-Akt, anti-phospho-ERK (T202/Y204), anti-ERK, anti-phospho-S6 (S240/244), anti-S6, anti-phospho-4E-BP1(S65) and anti-4E-BP1 (Cell Signaling Technology, Beverly, MA, USA). ImageJ (freeware) was used to compare the density of the bands and finish the densitometric analysis.

### In vivo Studies

Nude mice, 4 or 5-weeks-old, were purchased from the National Cancer Institute. They were acclimated for 1-2 weeks before they were subjected to any experimental procedures. BxPC-3 cells (1x10^6^) were injected subcutaneously (s.c.) into mouse flanks. Tumor volumes were measured in two dimensions (length and width) with calipers prior to treatment and twice a week once treatment was initiated. Mice were also weighed at these times, and weight changes were calculated by the following formulary: [1- (new weight/initial weight)] × 100. Tumor sizes were calculated by the standard formula of Tumor Size = Length x Width^2^ x 0.5. Mice that developed tumors reaching 100-150 mm^3^ in size were randomized into the following four groups with 8 mice in each group: vehicle, GDC0941, AZD6244, or the combination treatment. Both drugs were administered once or twice daily by oral gavage in a volume of 10 ml/kg body weight. Drugs were dissolved in a vehicle of 0.5% (w/v) methycellulose/0.2% Tween 20 for administration. The PI3K inhibitor was delivered daily at a final concentration of 50 mg/kg [14], while the MEK inhibitor was administered twice daily at a final concentration of 25 mg/kg [15]. Control animals were given an equivalent volume of 0.5% methycellulose/0.2% Tween 20 only, twice daily by oral gavage. The treatment duration was 18 days. If the mouse tumor diameter became larger than 15 mm during treatment or the mouse’s weight decreased by 20% compared to its initial weight, the mouse would be euthanized by CO_2_ overdose. All studies were carried out in accordance with the protocol approved by the Washington University Institutional Animal Care Facility (Protocol number: 20100114).

### Statistical Analyses

Cell viability data were represented as the mean ± standard deviation (SD). Cell apoptosis data and tumor growth experiments were represented as the mean ± standard error of the mean (s.e.m.). Kaplan-Meier curves were used for survival analyses. Statistical comparisons were made using the ANOVA (single factor) and t-test (paired two samples for means and unpaired t-test), as indicated. A p value of < 0.05 was considered statistically significant.

## Results

### Concurrent inhibition of PI3K and MEK has a synergistic effect on pancreatic cancer cell lines growth *in vitro*

**Figure 1:**
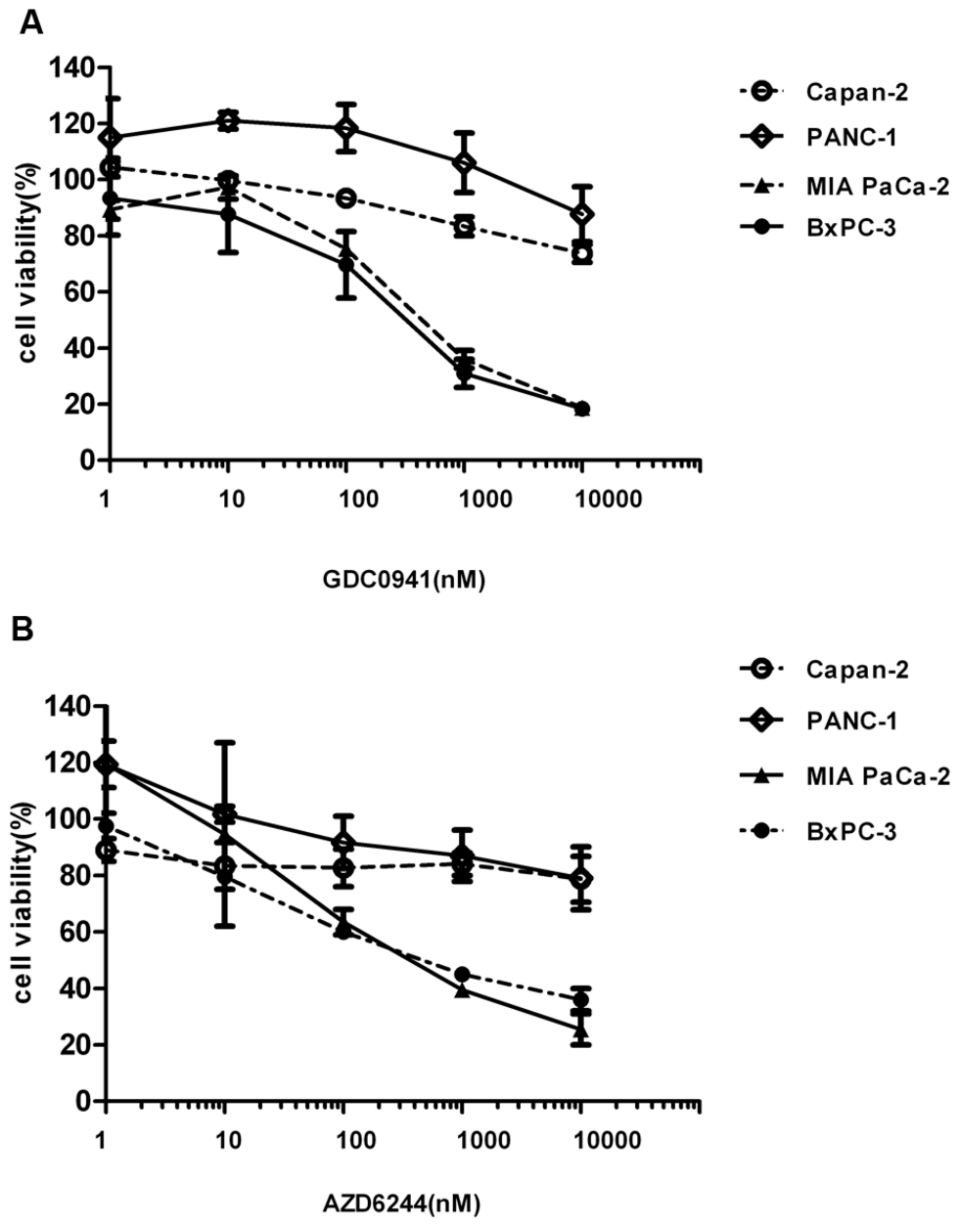
Cell viability effect of GDC0941 and AZD6244 in BxPC-3, MIA PaCa-2, PANC-1 and Capan-2 pancreatic cancer cell lines. Cells were treated with varying concentrations of GDC0941 (A) or AZD6244 (B) alone for 72 hours. Doses ranged from 1 nM to 10 µM.

To determine the anti-tumor activity of the PI3K and MEK inhibitors alone and in combination *in vitro*, four pancreatic cancer cell lines were selected for study. BxPC-3 is a KRAS wild type pancreatic cancer cell line, while the other three cell lines harbor the KRAS mutation. All four cell lines are not known to carry either PI3K or BRAF mutations [21]. The anti-proliferative effect of the PI3K or MEK inhibitor alone in BxPC-3, MIA PaCa-2, PANC-1 and Capan-2 cells was measured by Alamar Blue. Only the growth of BxPC-3 and MIA PaCa-2 cells was affected by GDC0941. The concentrations of GDC0941 resulting in 50% inhibition of cell viability (IC_50_) after 72 hours exposure were 376.4 nM in BxPC-3 cells and 754.6 nM in MIA PaCa-2 cells (Figure 1A). AZD6244 alone also suppressed cell growth with an IC_50_ value of 599 nM and 375 nM in BxPC-3 and MIA PaCa-2 cells, respectively (Figure 1B). The IC_50_ was not reached for PANC-1 and Capan-2 cells lines and as a result, these were considered to be resistant cell lines. We did observe a slight increase in PANC-1 cell growth with GDC0941at 1nM, 10nM, and 100nM, but changes were not statistically significant (control vs 1 nM, p=0.32, control vs 10 nM, p=0.17, control vs 100 nM, p=0.22). In addition, we compared control vs 1 nM AZD6244 in PANC-1 and MIA PaCa-2 cells, and no significant differences were observed (p=0.20, p=0.64, respectively)

**Figure 2:**
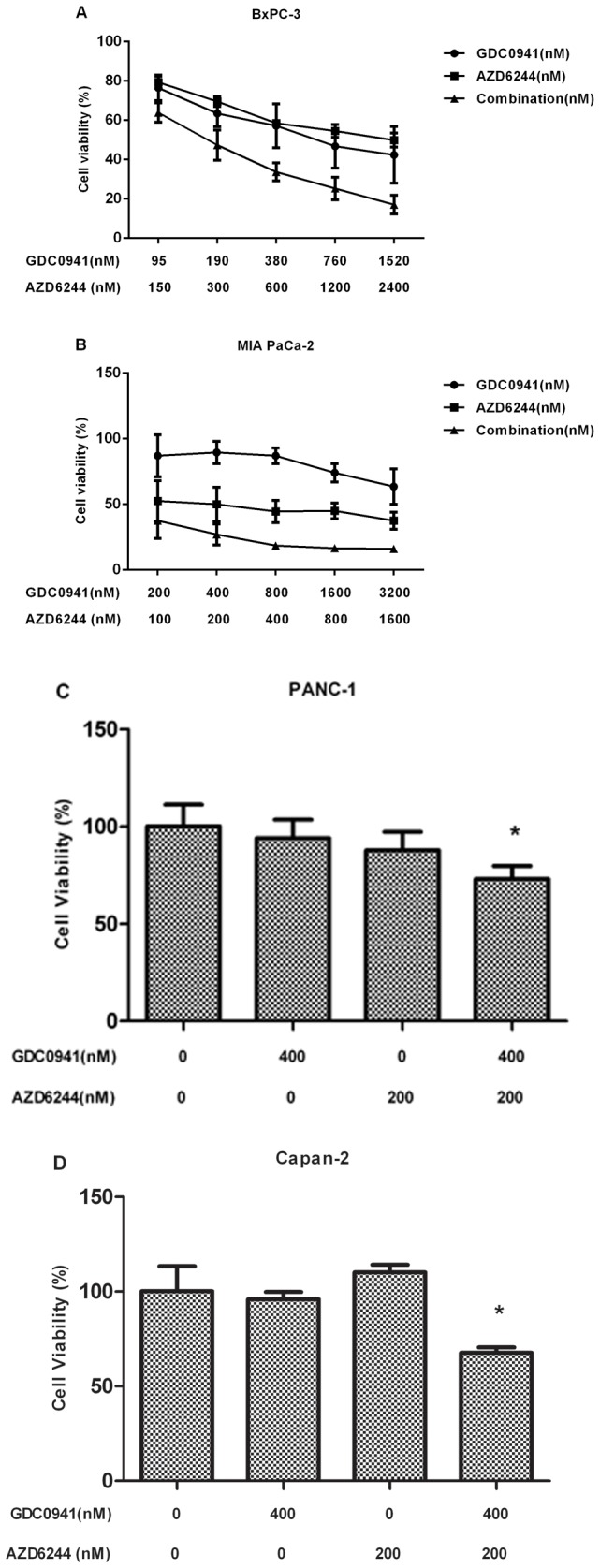
Synergistic effects of GDC0941-AZD6244 combination therapy on cell viability and proliferation. BxPC-3 (A) and MIA PaCa-2 cells (B) were treated with GDC0941 alone, AZD6244 alone or GDC0941-AZD6244 in combination. Results were analyzed according to the Chou-Talalay method [18]. The combination index (CI) values were calculated by using CalcuSyn software. PANC-1 (C) and Capan-2 cell line (D) were treated with GDC0941 at 400 nM, AZD6244 at 200 nM or combination. * p value <0.05 compared with control or single agent alone.

The anti-proliferative effect of combining a PI3K and MEK inhibitor was measured in BxPC-3 and MIA PaCa-2 cells by calculating the combination index (CI) according to the Chou-Talalay method (20) using a fixed dose ratio. Both GDC0941and AZD6244 were introduced to cell cultures at 0.25×, 0.5×, 1×, 2× and 4× their respective IC_50s_ in the BxPC-3 and MIA PaCa-2 cell lines. Cell growth in both cell lines was markedly decreased following combination treatment at multiple paired concentrations when compared with either single agent alone. Data were evaluated to get the CI under the corresponding effective dose (ED) in BxPC-3 and MIA PaCa-2 cell lines (Figure 2) by CalcuSyn software. For the BxPC-3 cell line the following CI values were obtained: 0.4101 (ED50), 0.0112 (ED75) and 0.0003 (ED90). For the MIA PaCa-2 cell line the CI values were 0.02052 (ED50), 0.0295 (ED75) and 0.0440 (ED95). The CI results suggested that GDC0941 and AZD6244 worked synergistically to produce an anti-proliferative effect in the BxPC-3 and MIA PaCa-2 cell lines (Figure 2A-B).

Interestingly, while GDC0941 or AZD6244 alone did not impact PANC-1 and Capan-2 cell growth, administration of these two drugs in combination mildly inhibited cell growth. Cell growth was reduced to 71.4% and 67.0% in the PANC-1 and Capan-2 cell lines, respectively, following administration of the drugs in combination (p<0.05, combination compared to untreated group or single agent alone) (Figure 2C-D).

### Concurrent PI3K and MEK inhibition induce apoptosis of pancreatic cancer cells lines *in vitro*

**Figure 3:**
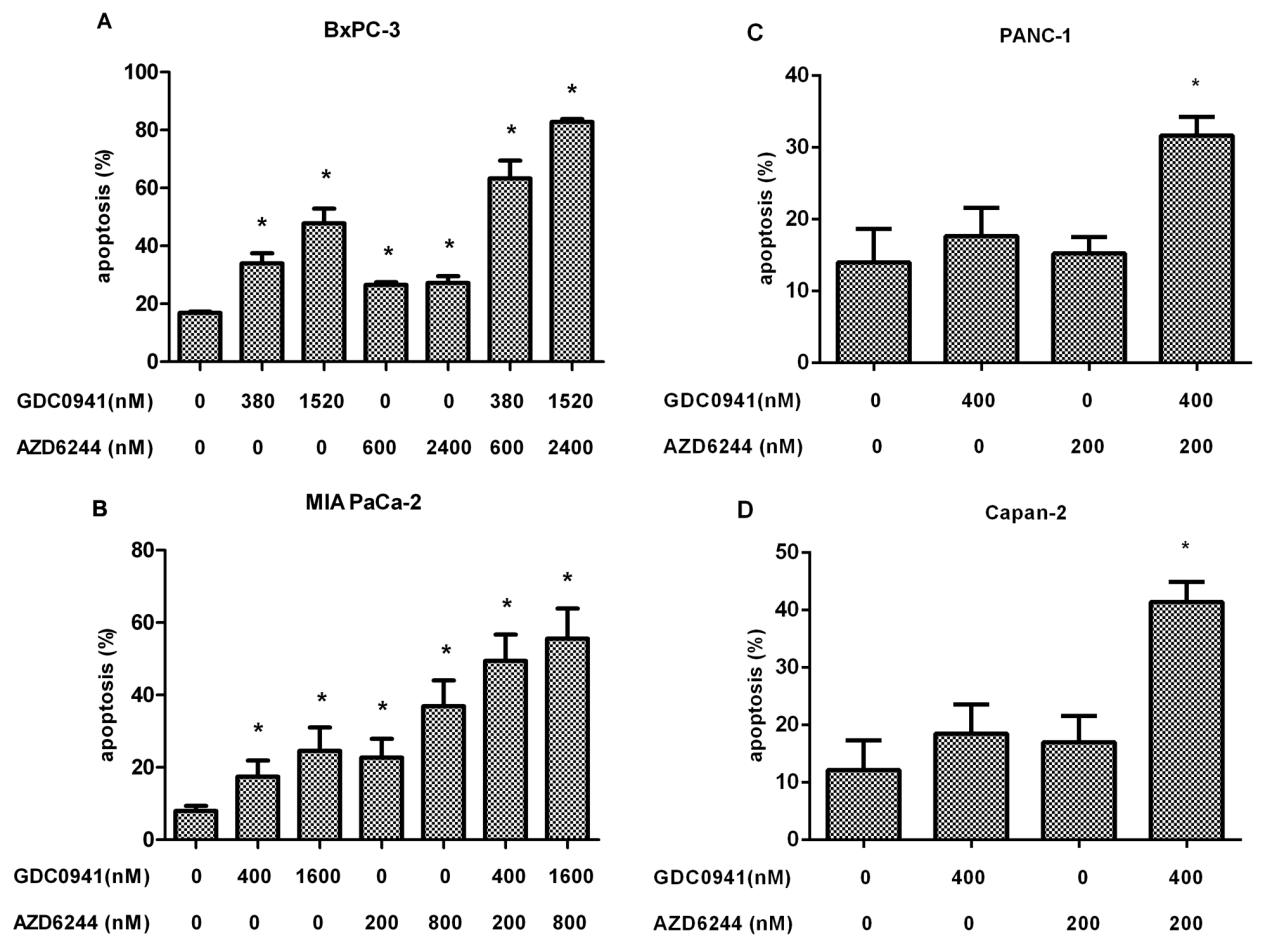
Effects of GDC0941 and AZD6244 therapy on cell apoptosis in pancreatic cancer cell lines. Pancreatic cancer cell lines were treated with GDC0941 alone, AZD6244 alone or GDC0941-AZD6244 in combination for 72 hours. Cell apoptosis was detected by flow cytometry. A, B: combinations vs. untreated groups (*p values <0.05), combinations vs. single agents (*p values <0.05), single agents vs. untreated groups (*p values <0.05). C, D: combinations vs. untreated groups (*p value <0.05) and combinations vs. single agents (*p value <0.05).

To determine the apoptotic effect of the combined therapy, two different concentrations of GDC0941 and AZD6244 were used alone and in combination. While the apoptosis rate of BxPC-3 cells at baseline was 17.0%, it increased significantly to 34.0% and 47.8% following administration of GDC0941 at 380 nM and 1,520 nM concentrations, respectively (p<0.05, GDC0941 alone vs. untreated group). AZD6244 alone at 600 nM and 2,400 nM increased the apoptosis rate of BxPC-3 cells to 26.5% and 27.2%, respectively (p<0.05, AZD6244 alone vs untreated group). A combination of GDC0941 and AZD6244 resulted in a much higher rate of apoptosis in BxPC-3 cells compared with the control group or inhibitor alone. The combination of GDC0941 at 380 nM and AZD6244 at 600 nM or the combination of GDC0941 at 1,520 nM and AZD6244 at 2,400 nM increased the BxPC-3 cell apoptosis rate to 63.3% and 82.8% respectively (p<0.05, combination vs. untreated group or single agent alone) (Figure 3A). The rate of apoptosis at baseline in MIA PaCa-2 cells was 8.0%, and it rose to 17.4% and 24.7% with GDC0941 administered at 400 nM and 1,600 nM concentrations, respectively (p<0.05, GDC0941 alone vs. untreated group). AZD6244 alone at 200 nM or 800 nM increased the apoptosis rate to 22.7% and 36.9%, respectively (p<0.05, AZD6244 alone vs. untreated group). The combination of GDC0941 at 400 nM and AZD6244 at 200 nM or the combination of GDC0941 at 1,600 nM and AZD6244 at 800 nM increased the MIA PaCa-2 apoptosis rate to 49.5% and 55.6%, respectively, and this was a statistically significant difference compared to the untreated group or single agent alone (p<0.05) (Figure 3B). For resistant cell lines PANC-1 and Capan-2, while neither agent alone had a significant impact on apoptosis, the combination of the PI3K and MEK inhibitor resulted in an apoptosis rate of 31.8% in PANC-1 cells; this was statistically significant compared to a 14.0% apoptosis rate in these cells without treatment or with a single inhibitor (p <0.05) (Figure 3C). Combining GDC0941 and AZD6244 also significantly increased the apoptosis rate in Capan-2 cells to 41.3%, compared to 12.2% without treatment or with single agent alone (p <0.05) (Figure 3D).

### Effects of PI3K and MEK inhibitions on cell signaling

**Figure 4:**
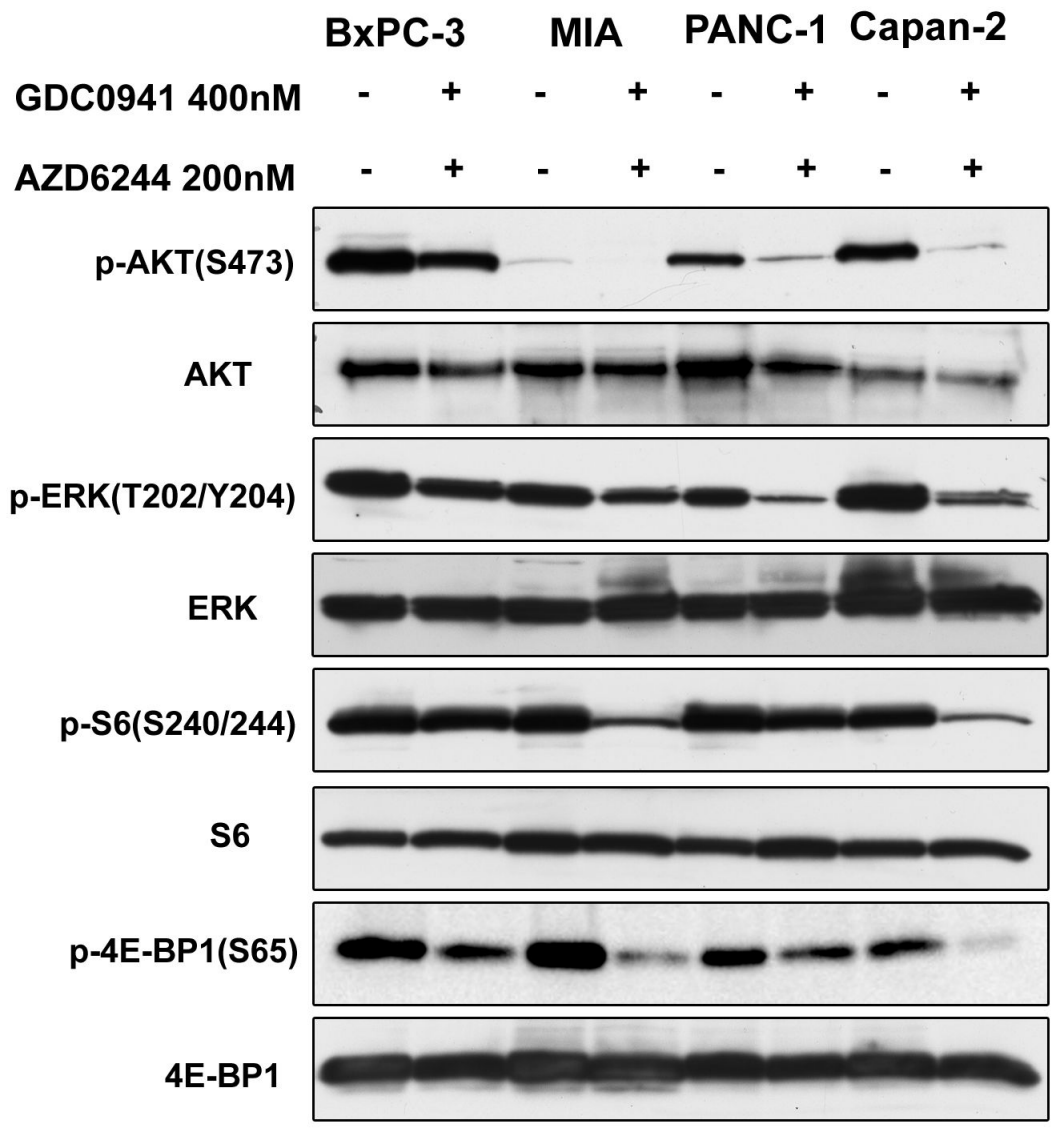
Effects of GDC0941-AZD6244 combination therapy on PI3K/AKT and MEK/ERK pathways. All four pancreatic cancer cell lines were treated with the GDC0941-AZD6244 combination for 24 hours, Cell lysates were then harvested to detect p-AKT (S473)/AKT, p-ERK (T202/Y204)/ERK, p-S6 (S240/244)/S6 and p-4E-BP1 (S65)/4E-BP1.

To assess the impact of both drugs on downstream effectors of the PI3K and MEK pathways, we used Western blot analysis to observe total protein expression and phosphorylation status. The total protein levels of ERK, S6 and 4E-BP1 remained unchanged after treatment with GDC0941 and AZD6244 in each cell lines (Figure 4). p-ERK, p-S6 and p-4E-BP1 appeared to be suppressed by GDC0941 and AZD6244 combination treatment. However, we observed changes in AKT expression following combination drug treatments, and densitometric analysis was used to quantify the expression levels. We used the ratio of p-AKT/AKT produced from each dose for comparison. After p-AKT/AKT levels of treatment groups were normalized to the ratio of the untreated group, the combination treatment was observed to suppress p-AKT levels by 90% in the BxPC-3 cell line, by 6% in the MIAPaCa-2 cell line, by 29% in the PANC-1 cell line, and by 8% in the Capan-2 cell line. The combination of both drugs reduced p-ERK (T202/Y204), p-AKT (S473), p-S6 (S240/244) and p-4E-BP1 (S65) expression compared with baseline in all cell lines tested. While p-AKT and p-ERK levels were differentially expressed in the four cell lines, our study showed that the baseline levels of p-AKT did not predict response to the PI3K inhibitor, nor did baseline p-ERK levels predict response to the MEK inhibitor.

**Figure 5:**
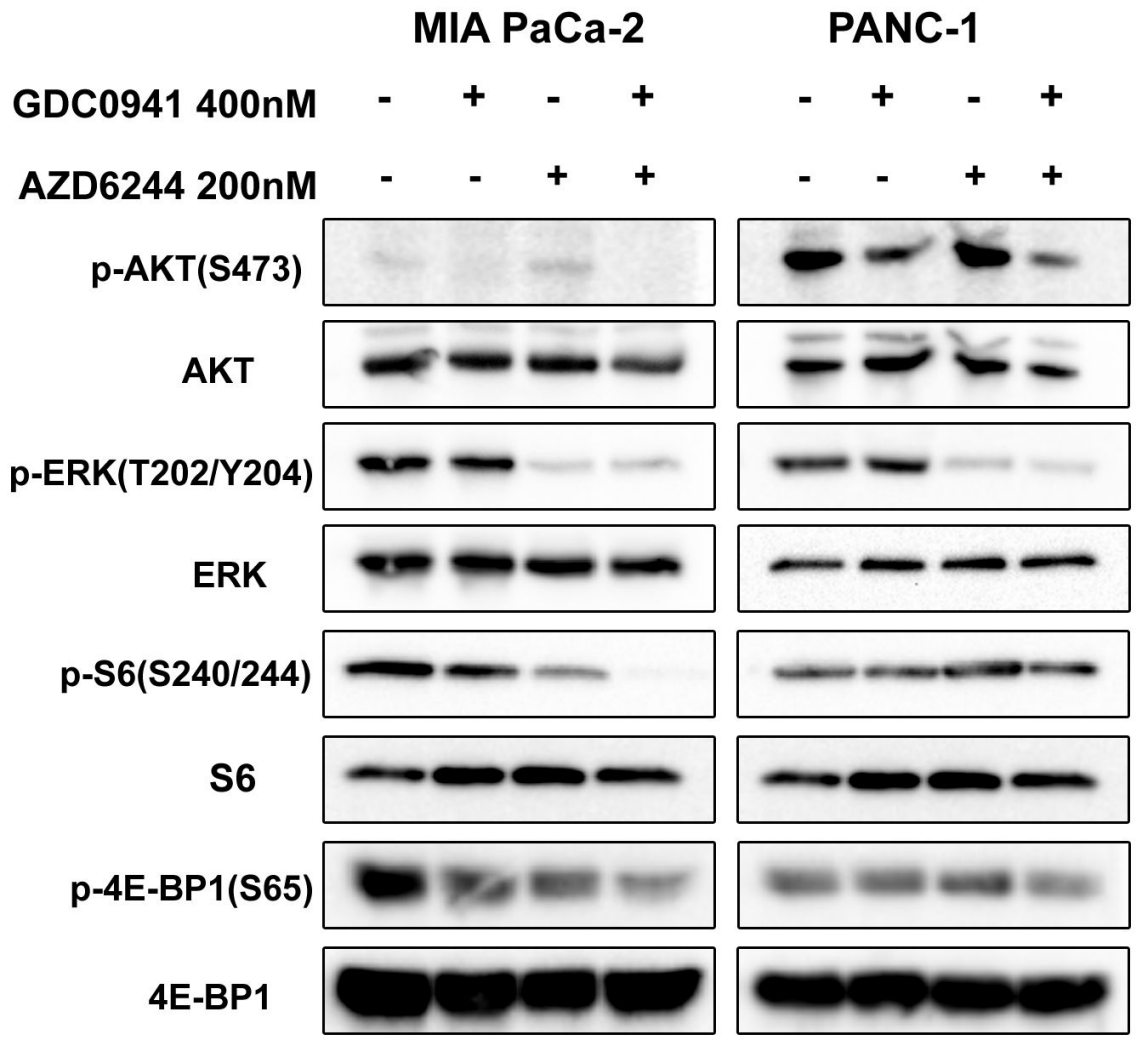
Effects of GDC0941 and AZD6244 on PI3K/AKT and MEK/ERK pathways of MIA PaCa-2 and PANC-1 cell lines. Both MIA PaCa-2 and PANC-1 cells were treated with 400 nm GDC0941, 200 nM AZD6244 or a combination at these doses for 24 hours. Cell proteins were then harvested to detect p-AKT(S473)/AKT, p-ERK(T202/Y204)/ERK, p-S6(S240/244)/S6 and p-4E-BP1(S65)/4E-BP-1.

To understand the phenotypic differences seen in the MIA PaCa-2 (sensitive to GDC0491 and AZD6244) and PANC-1 (resistant to GDC0491 and AZD6244) cell lines, both of which harbor the KRAS mutation, we examined the differences in their downstream effectors following GDC0941 and AZD6244 administration (Figure 5). In both cell lines, GDC0941 suppressed phosphorylation of AKT, AZD6244 decreased p-ERK levels, and the combination of the two drugs suppressed both p-AKT and p-ERK levels. GDC0941 and AZD6244 had a similar effect on AKT and ERK in the two cell lines. Interestingly, the impact from both inhibitors on p-S6 and p-4E-BP1 levels was, alternatively, cell line specific. For example, GDC0941 and AZD6244 alone and in combination markedly inhibited p-S6 and p-4E-BP1expression levels in MIA PaCa-2 cells, compared with the minimal suppression observed in PANC-1 cells (Figure 5). Neither GDC0941 nor AZD6244 alone suppressed p-S6 and p-4E-BP1 in PANC-1 cells, suggesting that both effectors may serve as biomarkers associated with treatment response. The expression levels of p-S6 and p- 4E-BP1 were significantly suppressed by the combination therapy in MIA PaCa-2 cells, and this was consistent with the cell line’s phenotypic responses toward combination treatment.

### Anti-tumor Effects of PI3K and MEK inhibitions *in vivo.*

**Figure 6:**
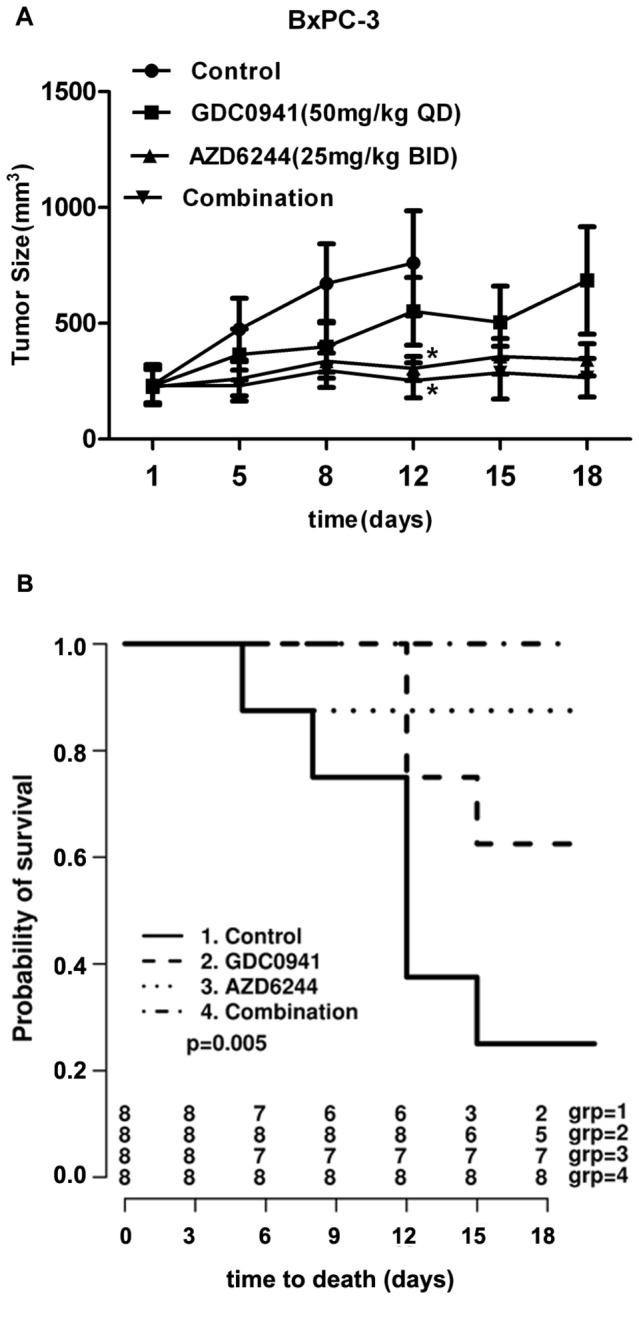
Effects of GDC0941 and AZD6244 on BxPC-3 xenograft model. A: 1×10^6^ BxPC-3 pancreatic cancer cells were injected s.c. into the right flank of female nude mice. Mice received vehicle (0.5% methylcellulose /0.2% Tween-20), 50 mg/kg GDC0941 QD, 25 mg/kg AZD6244 BID, or 50 mg/kg GDC0941 QD plus 25 mg/kg AZD6244 BID orally for 18 days. Data are presented as mean ± SE. *p values were determined by unpaired t-test. B: Kaplan-Meier survival curves of xenografts receiving vehicle, GDC0941 alone, AZD6244 alone and GDC0941-AZD6244 in combination.

To detect the effect of GDC0941 and AZD6244 on tumor growth *in vivo*, we used GDC0941, AZD6244, and a combination of GDC0941and AZD6244 to treat BxPC-3 xenograft mice for 18 days. Compared to the control (vehicle) group, tumor volumes decreased significantly in the AZD6244 and combination groups (p=0.037 and p=0.032, respectively) but not in the GDC0941group as compared to control (Figure 6A). Based on the Kaplan-Meier curves (Figure 6B), there was a statistically significance difference in survival among the four groups (p=0.005).

## Discussion

Although targeted therapy has become a mainstream approach for cancer treatment, the clinical development of targeted agents in pancreatic cancer has not been successful. Because of the high frequency of KRAS mutations in pancreatic cancer, KRAS has been directly targeted in pre-clinical and clinical trials, but results have been disappointing. In light of these challenges, research efforts have refocused on targeting the KRAS downstream pathways, PI3K and MEK. The benefit of blocking an individual pathway has been largely limited by the presence of a compensatory feedback loop between PI3K and MEK. For example, inhibition of the MEK pathway results in activation of the PI3K pathway [11], and PI3K activation mediates resistance to MEK inhibition [22]. To circumvent this compensatory feedback, concurrent blockade of the two pathways has been tested, and synergy in anti-tumor effects was detected, providing the rationale for phase I clinical trials. Moreover, early signs of clinical benefit have been reported in advanced cancer by a retrospective analysis on patients receiving agents that target both pathways [23].

In contrast to work in other types of tumors, preclinical evaluations of downregulating both pathways in pancreatic cancer have been limited [19,24]. Our study is important in several aspects. First, we have showed that the sensitivity of pancreatic cancer cell lines toward either PI3K or MEK inhibitors is not KRAS dependent. While differing in KRAS status, BxPC-3 and MIA PaCa-2 cells have wild type PI3K and PTEN, and both were sensitive to both inhibitors, which is consistent with previously published reports [22,25]. Secondly, our study showed that PI3K and MEK inhibition either alone or in combination can induce apoptosis. In the past, drugs targeting PI3K or MEK were thought to have more of a cytostatic effect, but recent report suggests that this effect is apoptotic [26] Thirdly, our study has demonstrated synergy in suppression of cell growth and induction of apoptosis in two sensitive cell lines (BxPC-3 and MIA PaCa-2) with the combination regimen. Moreover, mild inhibition in cell growth and induction of apoptosis were observed with the drug combination in resistant cell lines (PANC-1 and Capan-2). Although the degree of benefit from the combination treatment was modest for both resistant cell lines, further understanding of this benefit is warranted for this devastating disease. Our study supports a similar preclinical study in pancreatic cancer which showed that the treatment benefit of a MEK inhibitor was enhanced by an AKT inhibitor [24].

A crucial element of targeted therapy development is to determine molecular markers that predict the treatment response. PI3K pathway alterations including HER2 amplification, PI3KCA mutations or PTEN loss have been found to be associated with sensitivity to GDC0941 in breast cancer cell lines *in vitro* and *in vivo* [27]; however, the above genetic alternations are rarely present in pancreatic tumors [21]. Our study suggests that downstream p-AKT suppressed by GDC0941 does not predict cell sensitivity, nor does downregulated p-ERK predict sensitivity to AZD6244. This observation is consistent with previous reports [27].

To further understand the molecular events occurring after concurrent blockade in both KRAS mutated cell lines, we compared protein expression in the PANC-1 (resistant) and MIA PaCa-2 (sensitive) cell lines. These two cell lines differ in their phenotypic response to PI3K and ERK inhibitors, despite harboring similar major genetic alternations. Both cell lines are reported to contain KRAS mutations, p53 mutations, wild type P16 and wild type DPC-4 [21]. While in our study we demonstrated that suppression of p-AKT by the PI3K inhibitor and p-ERK by the MEK inhibitor were achieved in both cell lines, PANC-1 cells experienced only a minimal suppression of p-S6 and p-4E-BP1 when treated with either the PI3K or MEK inhibitor alone. These finding suggests that the PI3K or MEK inhibitors alone are able to suppress their immediate corresponding downstream mediators (AKT and ERK) in PANC-1 cells, but are not able to downregulate the further downstream mediators (p-S6 and p-4E-BP1). Both markers, therefore, are closely associated with sensitivity to both PI3K and MEK inhibitors. In a complex signaling network, a targeted agent’s capacity to inhibit the phosphorylation process of its downstream targets frequently does not translate into phenotypical changes. For example, Serra et al have reported the identification of a few genes which may promote cellular survival in the context of PI3K blockade, and among those genes, ribosomal S6 kinases RPS6KA2 (RSK3) and RPS6KA6 (RSK4) are being further validated for contributing to PI3K resistance *in vitro* and *in vivo* through attenuation of the apoptotic process and upregulation of protein translation [28]. Our observation resonates with emerging evidence that downstream cap-dependent translation may be a better indicator of response to these targeted agents [29,30].

4E-BP1 plays a major role in cap-dependent translation. It binds to the elF4E-mRNA cap complex to inhibit cap-dependent translation, and phosphorylated 4E-BP1 then falls out of the translation complex, so initiation of translation can begin [31]. mTORC1, an effector downstream of the PI3K pathway, can phosphorylate 4E-BP1 to initiate the translation process [32]. The crucial role of 4E-BP1 as a key effector of the AKT and ERK signaling pathways in tumors has been elegantly studied by She et al [29]. Moreover, high expression levels of 4E-BP1 have been found to have prognostic value in several tumor types [33-37]. Therefore, further exploration of targeting 4E-BP1 should be explored in the future for multiple tumor types, including, and especially, pancreatic cancer.

In summary, we have explored the benefit of concurrent pathway blockade by PI3K and MEK inhibitors alone and in combination for pancreatic cancer. Synergy in decreasing cell growth was observed and the effects were not KRAS dependent. Persistent phosphorylation of S6 and 4E-BP1 appeared to be associated with resistance to the PI3K and MEK inhibitors. Future investigations into the alternative mechanisms of 4E-BP1 phosphorylation and targeting of 4E-BP1 in pancreatic cancer are warranted.
