# Correction: Synergistic Effects of Concurrent Blockade of PI3K and MEK Pathways in Pancreatic Cancer Preclinical Models

**DOI:** 10.1371/journal.pone.0127365

**Published:** 2015-04-27

**Authors:** Hua Zhong, Cesar Sanchez, Dirk Spitrzer, Stacy Plambeck-Suess, Jesse Gibbs, Williams G. Hawkins, David Denardo, Feng Gao, Robert A. Pufahl, Albert C. Lockhart, Mai Xu, David Linehan, Jason Weber, Andrea Wang-Gillam

The third author’s name is spelled incorrectly. The correct name is: Dirk Spitzer. The correct citation is: Zhong H, Sanchez C, Spitzer D, Plambeck-Suess S, Gibbs J, Hawkins WG, et al. (2013) Synergistic Effects of Concurrent Blockade of PI3K and MEK Pathways in Pancreatic Cancer Preclinical Models. PLoS ONE 8(10): e77243. doi:10.1371/journal.pone.0077243
